# Psychological distress and resilience in patients with advanced cancer during the Covid-19 pandemic: the mediating role of spirituality

**DOI:** 10.1186/s12904-022-01034-y

**Published:** 2022-08-13

**Authors:** Luka Mihic-Gongora, Paula Jiménez-Fonseca, Raquel Hernandez, Mireia Gil-Raga, Vilma Pacheco-Barcia, Aránzazu Manzano-Fernández, Susana Hernando-Polo, Mónica Antoñanzas-Basa, María J. Corral, María Valero-Arbizu, Caterina Calderon

**Affiliations:** 1 Department of Medical Oncology, Hospital Universitario Central de Asturias, ISPA, Oviedo, Spain; 2 Department of Medical Oncology, Hospital Universitario de Canarias, Tenerife, Spain; 3 Department of Medical Oncology, Consorcio Hospital General Universitario de Valencia, Valencia, Spain; 4 Department of Medical Oncology, Hospital Central de la Defensa “Gómez Ulla”, Madrid, Spain; 5 Department of Medical Oncology, Hospital Universitario Clínico San Carlos, Madrid, Spain; 6 Department of Medical Oncology, Hospital Universitario Fundación Alcorcón, Madrid, Spain; 7 Department of Medical Oncology, Hospital Universitario Clínico San Carlos, Madrid, Spain; 8 Department of Clinical Psychology and Psychobiology, Faculty of Psychology, University of Barcelona, Passeig de la Vall d’Hebron, 171, 08035 Barcelona, Spain; 9 Department of Medical Oncology, Hospital Quirónsalud, Sevilla, Spain

**Keywords:** Advancer cancer, Palliative care, Disease outbreaks, Psychological distress, Resilience, End of life

## Abstract

**Background:**

The purpose of this study was to investigate the sociodemographic factors related to psychological distress, spirituality, and resilience, and to examine the mediating role of spirituality with respect to psychological distress and resilience in patients with advanced, unresectable cancer during the Covid-19 pandemic.

**Methods:**

A prospective, cross-sectional design was adopted. Data were collected from 636 participants with advanced cancer at 15 tertiary hospitals in Spain between February 2019 and December 2021. Participants completed self-report measures: Brief Resilient Coping Scale (BRCS), Brief Symptom Inventory (BSI-18), and Spiritual well-being (FACIT-Sp). Hierarchical linear regression models were used to explore the mediating role of spirituality.

**Results:**

Spirituality was significantly different according to the person’s age and marital status. Psychological distress accounted for 12% of the variance in resilience (β = − 0.32, *p* < 0.001) and spirituality, another 15% (β =0.48, *p* < 0.001). Spirituality acted as a partial mediator in the relationship between psychological distress and resilience in individuals with advanced cancer.

**Conclusions:**

Both psychological distress and spirituality played a role in resilience in cases of advanced cancer. Spirituality can help promote subjective well-being and increased resilience in these subjects.

## Introduction

Since the beginning of the coronavirus pandemic, more than 280 million cases of infection and more than five million deaths have been reported [1]. Cancer patients are at increased risk of developing COVID-19, severe SARS-CoV infection and consequent deterioration associated with their immunosuppressed state due to cancer and antineoplastic treatment [2, 3]. Fear of SARS-CoV-2 infection, difficulty in accessing medical care, fear of relapse or disease progression due to treatment delay or modification are more common concerns among individuals with cancer during this period shaped by the pandemic [2]. These apprehensions can be accompanied by greater psychological distress, increased anxiety and depression [3], causing them to be more preoccupied about dying, thereby aggravating their anguish and diminishing their quality of life [4].

Spirituality refers to how people search for and expression meaning and purpose in life and how they experience greater connection with themselves, others, and with the transcendental [5, 6]. According to Peterman et al. [5], spirituality can be understood as an essential element of health and overall well-being, and would therefore integrate dimensions of physical, psychological, and social health. It has attracted the attention of cancer research, in as much as it can help patients cope with the diagnostic and treatment processes better [6, 7], thereby contributing to reducing their psychological distress and enhancing their quality of life [7].

Resilience is another relevant aspect in the oncology population, as it can protect them from the detrimental effects of stress and adversity by softening the negative impact of the diagnosis; from treatment side effects, and from disease-related changes in lifestyle, and, in this way, improve their mental health and therapeutic outcomes [8, 9]. In one study of individuals with cancer who underwent hematopoietic stem cells, the more resilient participants reported less psychological distress and better quality of life than the less resilient ones [10]. In contrast, less resilient subjects report more anguish and depression [11, 12], even long after treatment [11].

Both spirituality and resilience have much to do with a person’s attempts to confront cancer and all the stressful events associated with it [11, 13]. There are no studies that probe the mediating role of spirituality between psychological distress and resilience in people with metastatic cancer. Consequently, this study analyzes the relationship between sociodemographic data and spirituality, resilience, and psychological distress and the mediating function of spirituality between these variables in subjects with advanced, unresectable cancer during the COVID-19 pandemic COVID-19. We hypothesize that, in these cases, spirituality will be a determinant in the relationship between psychological distress and resilience.

## Methods

### Design and patients

This is a multi-institutional, prospective, observational study funded by the Bioethics Group of the Spanish Society of Medical Oncology (SEOM). The study was conducted at 15 tertiary hospitals in Spain between February 2019 and December 2021, period coinciding with the COVID-19 pandemic. The study was performed in accordance with Good Clinical Practice guidelines and the Declaration of Helsinki. It was approved by research ethics board of each institution and classified by the Spanish Agency of Medicines and Medical Devices (AEMPS; Code: ES1402015).

Participants aged 18 years and older with histologically confirmed advanced, unresectable cancer and candidates for systemic treatment were consecutively enrolled. Individuals with any serious mental illness that prevented survey comprehension were excluded. Eligible patients were invited to participate in the study during the first visit to the medical oncology department for systemic treatment. Those who agreed to participate signed the consent form, were given instructions on how to fill in the written questionnaires, completed at home and handed them to the auxiliary staff at the next visit. Information was collected from clinical records or directly from the participants by medical oncologists. The database is managed via an online platform (www.neoetic.es).

### Measures

Participants completed the validated Spanish version of the following questionnaires.

The Brief Resilient Coping Scale (BRCS) [14] is a widely used questionnaire in cancer patients [15, 16] with a 4-item, and unidimensional outcome measure designed to capture to what extent an individual copes with stress in a resilient fashion [14]. Resilience has attracted the interest of the scientific community during the Covid-19 outbreak, as a protective factor in mental health. The items have a response format with five options, where 1 means the statement “does not describe you at all” and 5 means “it describes you very well”. The sum score varies between 4 to 20, the higher the score, the more resilience. Cut-off values of ≤13 and ≥ 17 are used to differentiate between low and high resilience scores [14]. Patients with a high resilience trait showed less distress and symptoms compared to those with low coping capacity [17]. Reliability for scale was 0.86 in the Spanish sample [16].

Spiritual well-being was appraised by the Functional Assessment of Chronic Illness Therapy-Spiritual Well-Being Scale (FACIT-Sp) [5, 18]. This instrument consists of 12 items scored on a five-point scale and contains two subscales, Meaning/Peace and Faith, and the total sum provide by the index of spiritual well-being that we have referred to as spirituality, simplifying the term. The higher the score, the greater the person’s wellbeing. Reliability for scale ranged from 0.85–0.86 in the Spanish sample [19].

Brief Symptom Inventory 18 (BSI-18) consists of 18 items divided into three dimensions (somatizations, depression, and anxiety), and a total score, the Global Severity Index (GSI), which summarizes the respondent’s overall emotional adjustment or psychological distress over the last 7 days [20]. Each item is rated on a 5-point Likert scale from 0 (not at all) to 4 (extremely). Cronbach’s alpha varied from 0.81 to 0.90 in Spanish sample [21].

Patient comorbidities were collected based on the International Classification of Diseases (ICD) diagnosis codes, and were categorized using the Elixhauser Comorbidity Index, which includes 29 diseases conditions. Elixhauser scores were calculated using the method proposed by van Walraven and colleagues [22].

### Data analysis

Data were statistically analyzed using the Statistical Package for Social Sciences (SPSS) for Windows 23.0 (SPSS Inc., Chicago, Illinois). All statistical tests were two-sided and the significance level was set at *p* < 0.05. Descriptive statistics for demographic and other variables were indicated by mean, standard deviation (SD), number (N) and percentage (%) as appropriate. T-tests and one-way ANOVA were used to compare differences in spirituality, resilience, and psychological distress between categorical groups. Eta-squared was reported as an indicator of the effect size of differences, with ranges between 0 and 1, with η^2^ ~ 01 for a small, η^2^ ~ 0.06 for a medium and η^2^ > 0.14 for a large effect size [23]. Pearson’s correlation was used to examine correlations between continuous variables. Hierarchical regression analysis was used to explore the mediating effects of resilience on the relationship between psychological distress and life satisfaction. According to Baron and Kenny’s technique on mediation [48], the following conditions should be met: (1) the independent variable (psychological distress) is significantly related to the dependent variable (resilience); (2) the independent variable (psychological distress) is significantly related to the mediator (spirituality); (3) the mediator (spirituality) is significantly related to the dependent variable (resilience), with the effect of the independent variable (psychological distress) on the dependent variable (resilience) upon adding the mediator (spirituality) to the model. Moreover, Sobel’s test was performed to estimate the mediation effect.

## Results

Of the 663 individuals recruited, 636 were eligible. A total of 27 were excluded (6 failed to meet the inclusion criteria; 5 met an exclusion criterion, and 16 had incomplete data). The mean age was 64.8 years (range, 24–89) and 53.1% (*n* = 338) were male. Most were married (84.4%), had a secondary education (50.8%), and all were retired or unemployed (100%). The most common cancers were bronchopulmonary (32.4%), digestive (39.1%), and breast (9.4%). Adenocarcinoma histology was the most frequent (62.1%) and most were stage IV (80.7%). The most common treatment was chemotherapy (53.1%), chemotherapy with targeted drug (14.2%), and chemotherapy with immunotherapy (12.1%). Estimated survival was less than 12 months in 26.3% of the sample. The characteristics of the study population can be found in Table 1.

**Table 1:** Comparison of mean total scores for psychological resilience (BRCS), psychological distress (BSI), and spiritual well-being (FACIT) according to baseline sample characteristics

Characteristics	N (%)	BRCS (mean ± SD)	BSI-18 (mean ± SD)	FACIT-Sp (mean ± SD)
**Sex**				
Male	338 (53.1)	14.5 ± 3.9	66.1 ± 7.1	36.5 ± 6.8
Female	298 (46.9)	14.0 ± 3.8	68.3 ± 7.3	36.4 ± 6.4
*p* value		0.080	**0.001**	0.719
**Age**				
≤ 50 years	56 (8.8)	14.6 ± 3.2	69.8 ± 7.4	33.6 ± 6.7
51–70 years	392 (61.6)	14.6 ± 3.7	66.6 ± 7.3	36.6 ± 6.5
> 70 years	188 (29.5)	13.4 ± 4.2	67.4 ± 7.3	37.4 ± 6.6
*p* value		**0.001**	**0.009**	**0.002**
**Marital Status**				
Married/ partnered	489 (76.8)	14.5 ± 3.8	66.8 ± 7.3	36.9 ± 6.3
Not partnered	147 (23.1)	18.8 ± 3.2	68.2 ± 6.9	34.9 ± 6.9
*p* value		0.447	0.120	**0.015**
**Educational level**				
Primary	313 (49.2)	13.8 ± 4.1	67.6 ± 7.3	37.0 ± 6.6
High school or higher	323 (50.8)	14.7 ± 3.6	66.7 ± 7.3	36.0 ± 6.7
*p* value		**0.006**	0.115	0.061
**Elixhauser comorbidities**				
≤ 4	214 (33.6)	14.1 ± 3.9	67.6 ± 7.4	36.1 ± 6.8
> 4	422 (66.4)	14.3 ± 3.8	66.9 ± 7.3	36.7 ± 6.5
*p* value		0.456	0.230	0.225

*Abbreviations*: *BRCS* Brief Resilience Scale, *BSI-18* Brief Symptom Inventory, *FACIT* Functional Assessment of Chronic Illness Therapy-Spiritual Well-Being Scale

Bold values indicate the significant at 5% level

Just over 30 % (30.5%) of the participants were found to be highly resilient copers. Patients > 70 years and those with a primary education scored lower on resilience than those ≤70 years and those with a higher education (*F =* 7.044, *p* = 0.001, η^2=^0.022; *F =* 7.471, *p* = 0.006, η^2=^0.012, respectively). Women displayed greater psychological distress than men (*F =* 14.985, *p* = 0.001, η^2=^0.023), as did subjects < 50 and > 70 years of age (*F =* 4.775, *p* = 0.009, η^2=^0.015). Participants < 50 years and those without a partner scored the lowest on spirituality (*F =* 6.093, *p* = 0.002, η^2=^0.019; *=*5.985, *p* = 0.015, η^2=^0.012, respectively).

### Correlations across variables

The mean, standard deviations of the variables, and Pearson correction analyses are presented in Table 2. The mean BRCS, BSI-18, and FACIT-Sp scores were 14.3 ± 3.8, 67.1 ± 7.3, and 36.5 ± 6.6, respectively. The results revealed that there were significant correlations across all psychological variables and that these correlations were in the direction expected. Psychological distress correlated negatively with resilience and spiritual well-being, while resilience correlated positively with spiritual well-being. Therefore, the first two conditions of Baron and Kenny’s technique were met in the present study.

**Table 2:** Correlations between BRCS, BSI, and FACIT-Sp scores

	Mean	SD	BRCS	BSI-18
BRCS	14.3	3.8	1	
BSI-18	67.1	7.3	−0.348**	1
FACIT-Sp	36.5	6.6	0.482**	−0.320**

*Abbreviations*: *SD* Standard deviation, *BRCS* Brief Resilience Scale, *BSI-18* Brief Symptom Inventory, *FACIT-Sp* Functional Assessment of Chronic Illness Therapy-Spiritual Well-Being Scale

**p < 0.001

### The mediating role of resilience in the relationship between psychological distress and spiritual well-being

**Fig. 1:**
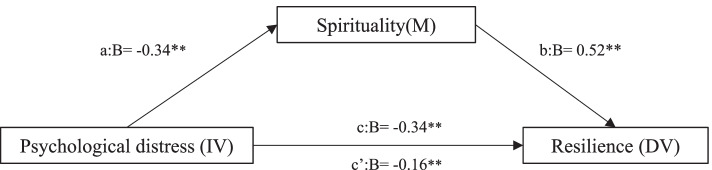
Mediation model for psychological distress, spirituality, and resilience, adjusted for age. a = direct effect of independent variable (IV) on mediator (M). b = direct effect of mediator on dependent variable (DV). c = direct effect of IV on DV. c’ = indirect effect of IV on DV. *p < 0.05. **p < 0.01

Hierarchical linear regression analyses to explore the mediating role of spirituality are represented in Fig. 1. After adjusting for age, the results reveal that psychological distress was negatively associated with spirituality and resilience (*β* = − 0.32, *p* = 0.001; *β* = − 0.34, *p* = 0.001, respectively), whereas spirituality correlated positively with resilience (*β* = 0.48, *p* = 0.001). Furthermore, spirituality mediated partially in the association between psychological distress and resilience, as the absolute value of its standardized regression coefficient (β) decreased from − 0.34 to − 0.21 (Sobel test, z = 6.835, *p =* 0.001).

## Discussion

To the best of our knowledge, this is the first study to explore spiritual well-being in individuals with advanced cancer during the COVID-19 pandemic and to scrutinize the mediating role of spirituality between psychological distress and resilience in this population. The results established that spirituality played a 12.1% mediating role and, as expected, found a negative correlation between psychological distress, spirituality, and resilience.

Many psychosocial studies have been conducted in patients with cancer in recent years [24]. Psychological distress and depression have been proven to be risk factors [12, 25] and spirituality to be protective in the development of depression [13, 26]. A systematic review of studies in cases of advanced cancer revealed that resilience is associated with spirituality, social support, the search for meaning, accepting their disease, positive attitude, and quality of life [8, 11]. During the COVID-19 pandemic, female breast cancer survivors who scored high on spirituality and resilience experienced less fear of recurrence, despite not receiving their usual medical follow-up [8, 11].

Despite the positive correlation between spirituality and resilience, they are considered to have their own, distinct characteristics [26, 27]. Recent research points toward spirituality potentially increasing resilience in different ways: favoring interpersonal relationships, as a source of strength and inner solace, or deceasing feelings of anger and social isolation [28, 29]. In this manner, spirituality could nurture resilience in patients with advanced cancer, but not vice versa, insofar as there can be resilient individuals without high levels of spirituality. This was found in the present study in which subjects > 70 years were not the most resilient despite exhibiting higher levels of spirituality. The greater frailty and vulnerability (comorbidities, lower functionality) and dependency, and poorer tolerance to cancer treatment of the elderly may account for this finding [30, 31]. Similarly, our results indicate that seniors (> 70 years) display less resilience and greater psychological distress. This is in line with earlier investigations that demonstrate that seniors have less resilience and more psychological issues like depression, attributable to their loneliness with less social support, lack of energy, and physical decline [17, 32]*.*

Our study reveals that, graphically, psychological distress is U-shaped; i.e., it is highest in patients ≤50 and > 70 years. This could be due to young adults finding their chances of achieving their life goals limited by their diagnosis of advanced cancer [33]. In seniors, psychological distress has been associated with them being physically weaker and suffering greater psychological affliction given the loss of significant people in their surroundings [29]. Our study also displayed greater psychological distress among women than men, which is in keeping with the literature that point toward females with cancer being more prone to psychological problems and suffering more from the repercussions on their family and milieu, given the organic, cosmetic, functional, and cognitive sequelae following their cancer diagnosis and treatment, as well as presenting more sexual problems [34, 35].

The study has a series of strengths and limitations. First, while the COVID-19 outbreak was a fundamental motivator in this study, it was designed before then and none of the variables collected was associated with the pandemic nor were infected individuals included, given that they had to overcome the disease in order to attend their oncology appointment. Secondly, given its cross-sectional nature, we were unable to draw causal relations across study variables. The findings of the current study should be confirmed by longitudinal cohort studies in the future. Third, all data were obtained through self-report questionnaires, which could introduce response bias. The participants may have underestimated or overestimated the relationship between the study variables. Fourth, the study did not seek to nor was it statistically powered to compare behavior of patients with different neoplasms; hence, the weight of tumor type has not been analyzed in the findings. It would be interesting to expand the sample to have representation of the different types of cancer and to stratify the analysis according to these. Finally, despite the fact that the sample is representative of the Spanish geography, any generalization of the results to other cultures and societies must be made with caution.

### Clinical implications

People with advanced, unresectable cancer find their life expectancy shortened and confront a situation in which spiritual concerns arise. Spirituality can help in the face of end-of-life despair, endowing the situation and one’s own existence with meaning and a sense of transcendence [11, 26]. The importance of spirituality notwithstanding, it is not easy for physicians to talk about the spiritual concerns of patients with advanced cancer.

Spirituality-based coping mechanisms can help to promote subjective wellbeing and greater resilience in cases of incurable cancer [36, 37]. Individual Meaning Centered Psychotherapy improves spiritual well-being and quality of life, reducing psychological distress in patients with advanced cancer [37]. Resilience, underpinned by spirituality, can help in the process of adapting to the disease and at the end of life [37].

Regardless of society’s secularization over the last 50 years, studies show that there is increased interest in spiritual growth and religious activity in older adults [26]. Including spirituality in interventions and the training of healthcare professionals who work with subjects with advanced cancer and in palliative care can contribute to maintaining and enhancing the resilience and wellbeing of patients and their caregivers [38, 39]. Moreover, finding meaning to life, reformulating the narratives of loss, and being a member of a community, such as a religious community, are some ways in which spirituality can bolster resilience and help people handle the challenges of the disease. In conclusion, spirituality can help promote subjective wellbeing and resilience in individuals with advanced cancer.

## Data Availability

The datasets generated during and analyzed during the current study are not publicly available for reasons of privacy. They are however available (fully anonymised) from the corresponding author on reasonable request.
